# Detection and Differentiation of Volatile Compound Profiles in Roasted Coffee Arabica Beans from Different Countries Using an Electronic Nose and GC-MS

**DOI:** 10.3390/s20072124

**Published:** 2020-04-09

**Authors:** Gancarz Marek, Bohdan Dobrzański, Tomasz Oniszczuk, Maciej Combrzyński, Daniel Ćwikła, Robert Rusinek

**Affiliations:** 1 Institute of Agrophysics Polish Academy of Sciences, Doświadczalna 4, 20-290 Lublin, Poland; 2 Pomology, Nursery and Enology Department, University of Life Sciences in Lublin, Głęboka 28, 20-400 Lublin, Poland; b.dobrzanski@ipan.lublin.pl; 3 Department of Thermal Technology and Food Process Engineering, University of Life Sciences in Lublin, Głęboka 31, 20-612 Lublin, Poland; tomasz.oniszczuk@up.lublin.pl (T.O.); maciej.combrzynski@up.lublin.pl (M.C.); 4 Rodzinna Palarnia Coffee and Sons, Boczna Lubomelskiej 4, 20-070 Lublin, Poland; daniel.cwikla@coffeandsons.pl

**Keywords:** volatile compound profiles, electronic nose, gas chromatography, roasted coffee beans, chemometrics

## Abstract

This paper describes the possibility of electronic nose-based detection and discrimination of volatile compound profiles of coffee from different countries roasted in a Gothot roaster under identical time and thermal regimes. The material used in the study was roasted *Arabica* coffee beans from Brazil, Ethiopia, Guatemala, Costa Rica, and Peru. The analyses were carried out with the use of the Agrinose electronic nose designed and constructed at the Institute of Agrophysics of the Polish Academy of Sciences in Lublin. The results of the volatile compound profile analysis provided by the Agrinose device were verified with the GC-MS technique. Chemometric tests demonstrated a dominant role of alcohols, acids, aldehydes, azines, and hydrazides in the coffee volatile compound profile. The differences in their content had an impact on the odor profile of the coffees originating from the different countries. High content of pyridine from the group of azines was detected in the coffee from Peru and Brazil despite the same roasting conditions. The results of the Agrinose analysis of volatile substances were consistent and correlated with the GC-MS results. This suggests that the Agrinose is a promising tool for selection of coffees based on their volatile compound profile.

## 1. Introduction

Coffee beans are one of the most expensive and valuable agricultural beans. Coffee is well known worldwide and is part of the culinary culture of many countries. In terms of material value, the world coffee market is the second largest after the crude oil market. Two of the several dozen known coffee species, *Coffea Arabica* and *Coffea Canephora* (*Robusta*), dominate on the coffee production and trade market and have considerable economic importance [1]. These two species differ in the shape of beans, climatic and environmental requirements, chemical composition, taste, and aroma [2]. Beverages made from roasted *Arabica* coffee beans are characterized by high acidity and fruity aroma [3], whereas coffee brewed from *Robusta* beans is stronger and bitter and contains more caffeine [4]. *Arabica* coffee is known for its mild and harmonious flavor, while the smell of *Robusta* is defined as earthy and raw. 

The chemical composition of coffee beans depends on the species, variety, and fruit ripeness as well as the environment, bean harvesting methods, and storage conditions. Both species, i.e., *Arabica* and *Robusta*, differ in the content of caffeine, trigoneline, lipids, chlorogenic acids, oligosaccharides, and *polysaccharides*. One of most important coffee attributes, i.e., the aroma, largely depends on the species, climatic and soil conditions, cultivation, and post-harvest storage. It evolves during roasting and depends on the conditions of this process [5]. Reactions of decomposition of non-volatile compounds contained in raw coffee, i.e., pyrolysis, caramelization, and Maillard reactions, yield the final aroma [6]. The volatile fraction in roasted coffee is a highly complex mixture of different classes of compounds with no typical coffee flavor. On the other hand, drinking coffee is associated with the caffeine sticking by man. Used in small amounts, caffeine raises the mood, as well as increases concentration. However, when the dose is increased, the effects do not become stronger and its effects change to the opposite. A person becomes irritable, anxious, and his mood changes to negative. The level of caffeine decreases as the degree of coffee roasting increases—lightly roasted coffee is the highest level of caffeine. The degree of coffee roasting is correlated with the amount of pyridines and pyrazines and with the type and origin of the coffee. The progress in chromatographic methods, in particular high-performance gas chromatography, and the use of mass spectrometers as detectors have contributed to identification of almost 850 chemical compounds from various groups, e.g.,: pyrazines, pyridines, pyrroles, carbonyl compounds, furans, phenols, oxazoles, thiophenes and thiazoles, thiols and other sulfur compounds, and carboxylic acids, in roasted coffee [7].

Coffee drinkers are divided into those who drink the beverage without paying attention to the quality of the coffee beans and those who choose coffee drinks based on espresso supplemented with water, milk, frothed milk, chocolate, etc. The different methods for preparation of coffee drinks have their supporters. This is often associated with the coffee making tradition in a given country: Turkish coffee, French press, drip, espresso, Italian coffee, instant coffee, aeropress coffee makers, etc. are distinguished [8,9]. Consumers from the first group use coffee produced by popular manufacturers, including ground coffee, coffee beans, capsules, or instant coffee. This group does not focus on high values of coffee taste and aroma. The other group comprises consumers that are more aware of the origin of coffee beans and choose coffees with a specific taste and aroma as well as high quality [10]. There is an increase in consumption of coffee and a growing number of consumers searching for new flavor sensations and choosing consciously the type of drink and the origin of the beans (country and region of origin, species, and variety as well as bean processing, roasting, grinding, and brewing methods). This necessitates development of instrumental methods for determination of coffee aroma to enable customers to distinguish original coffees from regions with the tradition of production of high-quality coffee beans from cheaper varieties grown in worse conditions but often offered dishonestly as noble coffee types [11,12]. 

There are many methods for assessment of aroma, e.g., organoleptic and instrumental methods, e.g., gas chromatography (GC-MS) and chemical determination of the composition [13]. In addition to these methods for evaluation of the aroma and chemical composition of biomaterials, electronic nose devices have been increasingly used in recent decades as fast, simple, and non-invasive tools for assessment of the quality of biological products. The dynamic development of the technology of manufacturing electrochemical transducers and techniques for analyses of large data sets has facilitated a wider use of the electronic nose for the analysis of volatile substances in many areas of life [14,15,16]. The term “electronic nose” defines an instrument consisting of a set of chemically sensitive sensors recognizing simple and complex odors and saving the information digitally in the memory of the device [17]. 

In the present study, the electronic nose was used for detection of the smells of roasted coffee beans and determination of their profile as well as correlation of the response with the presence of characteristic volatile compounds in coffee beans originating from different regions of cultivation and the possibility to recognize the compounds. The study was conducted with the use of the Agrinose device, whose measuring system consists of a matrix of eight metal oxide semiconductor (MOS) sensors. This inexpensive device is used for rapid detection of aromas. The results of the e-nose analyses were verified using the more accurate but more laborious and expensive GC-MS technique for analysis of volatile substances.

## 2. Materials and Methods

### 2.1. Materials

The following *Arabica* coffee beans produced in five important production regions were used in the study: Costa Rica—La Pastora, Ethiopia—Sidamo, Brazil—Santos, Peru—El Palto Organic, Guatemala—Comal, and Ethiopia—Adado. The Costa Rican coffee originated from the ‘Caturra’ variety grown in the San Marcos de Tarrazu region in Costa Rica. The Ethiopian ‘Heirloom’ coffee beans were produced in the Sidamo region. The Santos coffee beans represented the ‘Bourbon’ variety grown in lowland areas in Brazil. The El Palto brand was the ‘Caturra’ variety, which is native to the Amazon Andes of Northern Peru. The Comal brand originated from the ‘Bourbon’ variety grown in the area of San Pedro Necta in Guatemala.

Coffee beans were roasted in a Gothot roaster. The final parameters of the roasting process were identical for all coffee types. The total roasting time was 12 min 30 s, with the drying phase—6 min, the initial roasting phase—4 min, the development phase—2 min 30 s. The batch of beans loaded into the roaster was identical for all brands, i.e., 10 kg.

### 2.2. Electronic Nose

The Agrinose device designed and constructed at the Institute of Agrophysics of the Polish Academy of Sciences in Lublin was used in the study [17]. It has a matrix of eight MOS sensors (TGS2600—general air contaminants, hydrogen and carbon monoxide; TGS2602—ammonia, hydrogen sulfide, high sensitivity to VOC, and odorous gases; TGS2603—odors generated from spoiled foods; TGS2610—LP gas, butane; TGS2611—natural gas, methane; TGS2612—methane, propane, and butane; TGS2620—solvent vapors, volatile vapors, alcohol; AS–MLV-P2—CO, butane, methane, ethanol, hydrogen. Specifically designed for volatile organic compounds). Agrinose was previously used to evaluate bread spoilage, rancidity of edible oils, and seed spoilage and for control of baking. In comparison with the previous studies, the device was modified by replacing sensor TGS2611 E00 (sensitivity to natural gas) with sensor TGS2603 for detection of food spoilage, which significantly improved the sensitivity of the device in identification of VOCs in biological materials [16].

**Figure 1:**
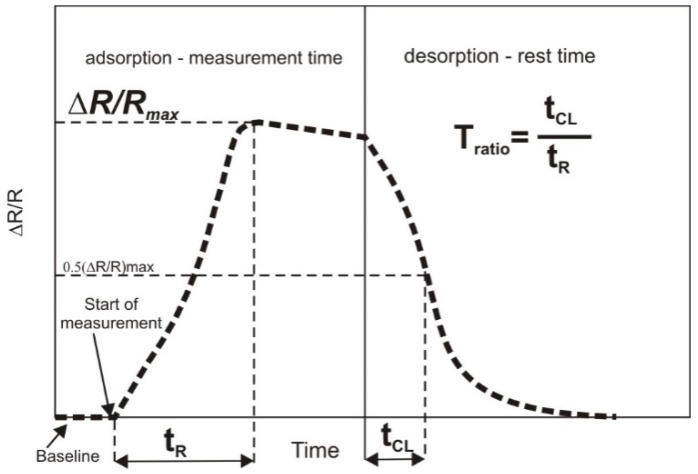
Scheme of a typical sensorgram for metal oxide semiconductor (MOS) sensors with a ratio of reaction times of *T_R_* and *T_CL_* marked on the graph [14].

In contrast to common descriptions of the odor profile, a new three-parameter method for generation of the smellprint was used in the present study (Figure 1). To date, the maximum sensor response Δ*R/R_max_*, i.e., the maximum value of the change in e.g., the resistance of MOS or conducting polymer (CP) sensors, has been most commonly used for generation of smellprints. The authors developed a new method for generation of smellprints based on two additional parameters [15,17]: the time until achievement of the maximum response—the response time *T_R_* and the cleaning time *T_CL_*, which is the time of removal of molecules from the sensor’s active surface, i.e., the time from achievement of the maximum response Δ*R/R_max_* to half of its value. The parameters established in the study were dependent on the type of volatile substances contained in the odor profile and the intensity of emissions of compounds. A new odor intensity factor was used, i.e., the ratio of the reaction times *T_ratio_* (Figure 1). Its physical interpretation is based on the assumption that the greater the value is than zero, the more intense the interaction of volatile molecules with the active surface is.

### 2.3. Gas Chromatography-Mass Spectrometry (GC-MS) Analysis

The GC-MS analyses were carried out using a Trace GC Ultra gas chromatograph (ThermoFisher Scientific, USA) coupled with an ITQ 1100 mass spectrometer (ThermoFisher Scientific, USA) following the procedure described by [18]. Volatile compounds were collected from the headspace by solid phase micro-extraction (SPME) [19]. SPME fiber 50/30 um Divinylbenzene/Carboxen/Polydimethylsiloxane (DVB/CAR/PDMS), Stableflex (2 cm) 24Ga (Sigma Aldrich, Poland) was used for the chromatographic analyses. The fiber with the adsorbent was placed for 30 min in a measurement chamber with a mixture of volatile organic compounds emitted by the coffee beans through a special valve. Then, it was transferred into a GC injector for 5 min for desorption of volatile organic compounds. The injection port equipped with a 0.75-mm i.d. liner was maintained at 250 °C in the splitless mode. A Zebron ZB-5Msplus Capillary GC 30 m × 0.25 mm × 0.25 um capillary column was used in the analysis. The following temperature program was established: initial temperature of 60 °C for 5 min, from 60 to 250 °C at 5 °C/min, from 250 to 270 °C at 10 °C/min, and final temperature maintained for 5 min. A constant helium flow rate was kept at 2.2 mL/min. The temperature of the transfer line and the ion source was 280 °C. The electron ionization (EI +) mode with an electron energy value of 70 eV was applied. The mass spectrometer collected data in the full scan mode (scan ranges: 35–390). The procedure was also described in [16].

### 2.4. Chemometrics and Statistical Analysis

The analysis of variance, simple correlations, and principal component analysis were carried out at the significance level α = 0.05 using Statistica software (version 12.0, StatSoft Inc., USA). Five coffee bean groups were analyzed: Brazil, Ethiopia, Guatemala, Costa Rica, and Peru. Tukey tests (one-way ANOVA, Statistica version 12.0, StatSoft Inc., USA) at the significance level of α = 0.05 were used. The PCA analysis determined the relationship between the sensor response Δ*R/R_max_*, *T_R_*, and *T_CL_* for the eight sensors used in the study and the volatile compounds. The optimal number of the principal components obtained in the analysis was determined based on the Cattel criterion. A data matrix with 5 rows and 55 columns was constructed to assess the ability of the Agrinose device to describe the odor profiles of the single-origin coffees. The input matrix was scaled automatically.

## 3. Results and Discussion

### 3.1. GC-MS Analysis

**Figure 2:**
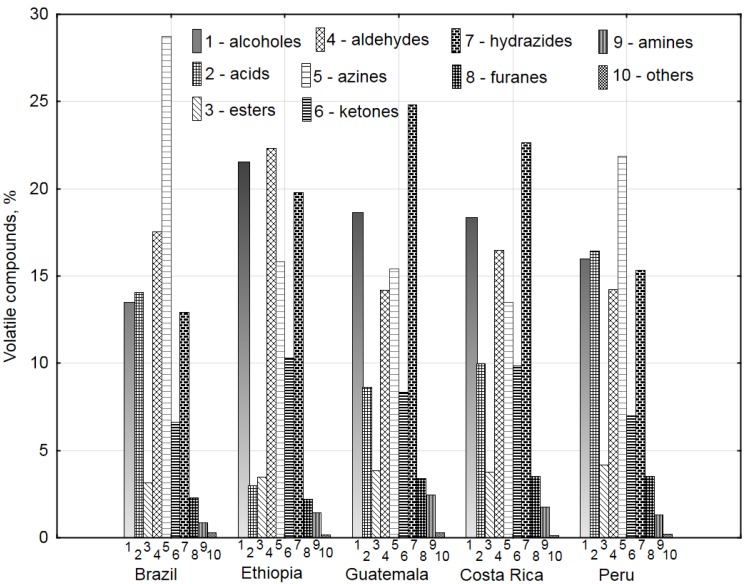
Percentage share of groups of volatile substances for all coffees beans after roasting.

The analysis of chromatograms generated for the individual samples allowed determination and assignment of over 99% of compounds to the main chemical groups (Table 1) constituting the odor profile of the *Arabica* coffees [11]. Figure 2 presents a composite graph of the percentage of the chemical compounds present in the coffees. The Wiley 138 library with the highest quality of match was used to identify the compounds [16]. The compounds were combined into the main chemical groups of VOCs and presented in percentages in the graphs. Alcohols, acids, aldehydes, azines, hydrazides, and ketones were the most abundant (Figure 2).

The Brazilian Santos coffee contained almost 30% of aromatic compounds, mainly represented by azines. The simplest compound in this group was pyridine [20,21] (28.7% content). Another group of compounds that contributed to the odor profile of the Santos coffee contained aldehydes, which accounted for about 17.5%. Alcohols, acids, and hydrazides represented approximately 12–13%, and ketones constituted approximately 7% of volatile compounds. The largest contribution to the smell profile of the Ethiopian Sidamo coffee was ascribed to aldehydes, alcohols, hydrazides, and azines, which in total accounted for approximately 80% in the odor profile; in turn, ketones constituted ca. 10%. Two *Arabica* coffees, i.e., Comal from Guatemala and La Pastor from Costa Rica, were characterized by a similar percentage of hydrazides (22–25%), alcohols (18%), azines and aldehydes (14–17%), and ketones (8–10%), respectively. The El Palto Organic *Arabica* coffee from Peru had similar contents of five groups of compounds: from 15 to 22% of alcohols, acids, aldehydes, azines, and hydrazides and 7% of ketones.

The data shown in Figure 2 and Table 1 suggest that coffee aroma is generated by a combination of many chemical compounds containing various functional groups [13,20]. The complex mechanisms of formation of the compounds are associated with many chemical reactions (e.g., degradation of sulfur amino acids, phenolic acids, fats, sugars, and pigments, Strecker degradation, etc.) [7]. However, aroma and taste are primarily generated via the Maillard reaction between sugars and amino acids [6]. 

The coffees from Brazil and Peru contained the highest content of pyridine, i.e., 28.7% and 21.9%, respectively. In the other three coffee brands, pyridines accounted for 13–15%. This compound is perceived as a bitter vegetable odor. In turn, aldehydes, ketones, esters, alcohols, and acids, i.e., compounds from the group of furans, were abundant in all coffee species. They are characterized by the ethereal smell of grass and hay. The coffee aroma also contained compounds with a pyrazine ring, which have a bittersweet aroma (Table 1). 

The differences in the contents of volatile compounds were related to the different conditions of coffee growth and harvest, interspecies differences, and pre-roasting [7] storage conditions [22]. They did not depend on the degree of roasting [21], as the final parameters of the roasting process were identical for all coffee types.

**Figure 3:**
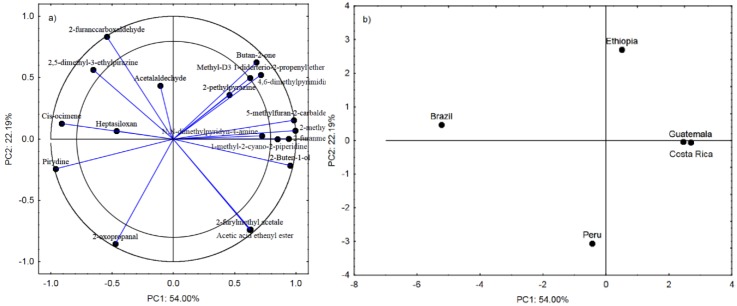
Loading plot (**a**) and score plot (**b**) of the principal components analysis carried out on the analytical data of the of the nineteen chemical compounds detected in all coffees from Brazil, Ethiopia, Guatemala, Costa Rica, and Peru.

Figure 3a shows the projection of the variables on the PC1 and PC2 planes. Chemical compounds located within the two circles have a strong effect on the possibility to determine the country of coffee origin [23]. Compounds in the negative quadrant of principal component PC1 differentiate the Brazilian and Peruvian coffee from the others. Noteworthy, pyridine and 2-oxoproponal (compounds located in the PC1 and PC2 negative quadrant) are most abundant in the Brazilian and Peruvian coffees. In turn, compounds on the right side of principal component PC1 well describe the *Arabica* species from Ethiopia, Guatemala, and Costa Rica (Figure 3b). The higher content of butan-2-one, 2-methylpirimidine, and 4.6-dimethylpyrimidine in the odor profile of the three coffees in comparison with the coffees from Brazil and Peru should be highlighted. Pyrimidines are organic volatile compounds from the group of heterocyclic aromatic compounds with a structure similar to pyridine.

They are responsible for an unpleasant bitter smell as well as the aroma of hazelnuts, vegetable butter, and caramel. In general, their amount in coffee is correlated with the degree of roasting [21], although its level in the present study was the same in all coffee types.

**Figure 4:**
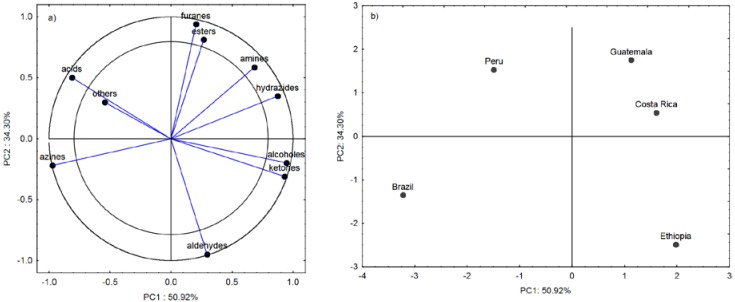
Loading plot (**a**) and score plot (**b**) of the principal components analysis carried out for ten chemical groups detected in all coffees from Brazil, Ethiopia, Guatemala, Costa Rica, and Peru.

Figure 4a shows the projection of the variables of the major groups of chemical compounds on the PC1 and PC2 plane. As in the case of the volatile compounds, all chemical groups except “others” (Figure 2) differentiated coffee according to the origin. The group of azines and acids were the most abundant volatile substances in the coffees from Brazil and Peru. This relationship is described by principal component PC1 (Figure 4a,b). In turn, the higher contents of alcohols, ketones, and hydrazides detected in the volatile compound profiles of the coffees from Ethiopia, Guatemala, and Costa Rica differentiated these beans from the Brazilian and Peruvian coffees by the first principal component as well. A similar relationship for the analyzed volatile chemicals is shown in Figure 3a,b.

The chemometric analysis indicates that the coffee species maintain their specific odor profile, which is usually determined by four dominant groups of volatile compounds [24]. 

### 3.2. Electronic Nose and Analysis

The results of the comparative analysis carried out with the use of the Agrinose device are consistent with those of the chemometric analysis, which clearly showed differences in the coffee odor profiles [23]. The MOS sensors used in the matrix (AS-MLV-P2, TGS2600, TGS2602, TGS2603, TGS2610, TGS2611, TGS2612, and TGS2620) proved to be suitable for discrimination of coffee aromas. The obtained parameters are presented in Table 2. Tukey tests (one-way ANOVA, Statistica version 12.0, StatSoft Inc., USA) at the significance level of α = 0.05 were used to comparison the results presented in Table 2. For most of the examined coffee bean species, significant differences were found between the obtained parameters. Only for Ethiopia and Peru coffee bean, did the analysis not show significant differences for Δ*R/R_max_* parameters obtained with AS-MLV-P2 and TGS 2612 sensors and *T_ratio_* obtained with TGS 2602 and TGS 2603 sensors. In addition, in the case of Ethiopia and Guatemala coffees there was no significant difference for the *T_ratio_* parameter obtained from TGS 2611 sensor. The highest Δ*R/R_max_* parameter values were obtained for Brazil coffee and the smallest for Guatemala coffee. High content of pyridine from the group of azines was detected in the coffee from Brazil. The one coffee with the smallest content of pyridine was detected in the coffee from Guatemala (Table 1). The analysis of Agrinose reaction results with volatile coffee substances was correlated with the GC-MS results (Table 2).

**Figure 5:**
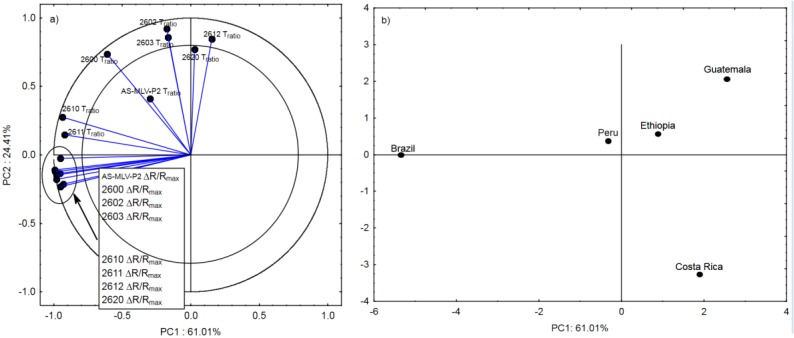
Loading plot (**a**) and score plot (**b**) of the principal components analysis carried out for sixteen sensor readings and types of coffee from Brazil, Ethiopia, Guatemala, Costa Rica, and Peru.

The first principal component PC1 indicates that the electronic response Δ*R/R_max_* parameter for all eight sensors and *T_ratio_* for the TGS2600, TGS2610, and TGS2611 sensors successfully discriminated the Brazilian coffee from the others, especially from the coffees from Ethiopia, Guatemala, and Costa Rica. The Peruvian coffee had a similar composition of volatile groups to the coffee from Brazil, which consequently located it on the left side of the first principal component PC1, as in the chemometric analysis. The odor profile of the Brazilian and Peruvian coffees was characterized by the highest content of azines, which include pyridine, responsible for the characteristic bitter smell. Approximately 15 compounds containing a pyridine ring were detected in the aroma profile of the coffees [24]. Its content in the aroma profile was indicated by the negative PC1 values. The lowest levels of this compound were detected in the coffees from Costa Rica and Guatemala. Consistent results for this parameter were obtained in the e-nose and chromatographic analyses of volatile compounds (Figure 3b, Figure 4b and Figure 5b).

## 4. Conclusions

The investigations demonstrated that the degree of roasting and the type of device used did not alter the individual aromatic properties of the analyzed coffees. The volatile compound profiles and the content of their main groups were specific to each coffee type and were associated with the conditions of growth, harvesting, and storage. The study showed a dominant role of alcohols, acids, aldehydes, azines, and hydrazides in the aromas of all coffees. As shown by the chemometric analysis, the high content of pyridine from the group of azines differentiated the types of coffee, which was especially evident in the case of the Brazilian coffee. The analysis of volatile compounds carried out with the use of the Agrinose yielded consistent results to those obtained with GC-MS. It should be concluded that the quick and low-cost analysis of volatile substances in coffee using an electronic nose with a matrix of MOS sensors is a reliable tool for assessment and classification of coffee types.

## Figures and Tables

**Table 1:** Chemical compounds detected in all *Arabica* coffees from: Brazil, Ethiopia, Guatemala, Costa Rica, and Peru.

No.	Name of Compounds	R_time_ *	Country of Origin				
				Brazil, %	Ethiopia, %	Guatemala, %	Costa Rica, %	Peru, %
1	2-Buten-1-ol	1.10	1.7	2.1	2.5	2.3	2.2
2	2-oxopropanal	1.17	14.1	3.0	8.6	10.0	16.4
3	Methyl-D3 1-diderterio-2-propenyl ether	1.35	6.3	11.4	8.3	7.7	6.1
4	Acetalaldechyde	1.45	4.9	8.5	3.4	4.9	5.9
5	Pirydine	1.76	28.7	15.8	15.4	13.5	21.9
6	Butan-2-one	2.31	6.6	10.3	8.3	9.9	7.0
7	2-methylpirimidine	2.54	2.9	9.3	10.8	10.5	7.7
8	2-furanccarboxaldehyde	2.64	10.9	10.6	7.1	7.8	5.7
9	2-furanmethanol	2.92	5.5	8.1	7.9	8.4	7.7
10	Acetic acid ethenyl ester	3.16	3.2	3.5	3.9	3.8	4.2
11	4.6-dimethylpyrimidine	4.25	5.5	6.2	7.6	6.8	4.4
12	2-pethylpyrazine	4.35	2.5	2.4	3.3	3.1	1.8
13	Cis-ocimene	4.87	0.1	0.0	0.1	0.1	0.0
14	5-methylfuran-2-carbaldehyde	5.78	1.7	3.2	3.7	3.8	2.6
15	2-furylmethyl acetale	6.91	2.3	2.2	3.4	3.5	3.5
16	N.N-dimethylpyridyn-4-amine	7.06	1.4	1.6	2.8	2.0	1.5
17	1-methyl-2-cyano-2-piperidine	7.23	0.9	1.4	2.5	1.8	1.3
18	2.5-dimethyl-3-ethylpyrazine	9.73	0.6	0.3	0.3	0.1	0.0
19	Heptasiloxan	34.36	0.3	0.2	0.3	0.1	0.2
Sum, Σ (%)			100	100	100	100	100

*—retention time.

**Table 2:** The mean values of the obtained parameters using the Agrinose device and the results of the significance difference analysis carried out using the Tukey test.

Country of Origin					
Parameter	Brazil	Ethiopia	Guatemala	Costa Rica	Peru
2602 Δ*R/R_max_*	3.17	2.22	1.17	1.88	1.98
SD	0.06	0.04	0.02	0.04	0.04
AS-MLV-P2 Δ*R/R_max_*	2.13	1.33 *	0.57	0.86	1.35 *
SD	0.04	0.03	0.01	0.02	0.03
2603 Δ*R/R_max_*	1.77	1.36	0.65	1.05	1.11
SD	0.04	0.03	0.01	0.02	0.02
2612 Δ*R/R_max_*	0.30	0.05 *	0.02	0.07	0.06 *
SD	0.01	0.01	0.01	0.01	0.01
2610 Δ*R/R_max_*	0.58	0.28	0.14	0.22	0.22
SD	0.01	0.01	0.01	0.01	0.01
2611 Δ*R/R_max_*	0.49	0.26	0.12	0.20	0.17
SD	0.01	0.01	0.01	0.01	0.01
2620 Δ*R/R_max_*	1.12	0.54	0.25	0.42	0.40
SD	0.02	0.01	0.01	0.01	0.01
2600 Δ*R/R_max_*	1.08	0.50	0.27	0.47	0.44
SD	0.02	0.01	0.01	0.01	0.01
2602 *T_ratio_*	2.19	1.46 *	1.80	1.38	1.48 *
SD	0.04	0.03	0.04	0.03	0.03
AS-MLV-P2 *T_ratio_*	2.29	6.15	2.57	1.27	3.11
SD	0.05	0.12	0.05	0.03	0.06
2603 *T_ratio_*	2.33	1.50 *	1.80	1.34	1.50 *
SD	0.05	0.04	0.04	0.03	0.03
2612 *T_ratio_*	2.35	2.58	3.00	1.40	2.82
SD	0.05	0.05	0.06	0.03	0.06
2610 *T_ratio_*	8.80	3.20	3.00	1.32	3.39
SD	0.18	0.06	0.06	0.03	0.07
2611 *T_ratio_*	8.00	3.00 *	3.00 *	1.44	3.21
SD	0.16	0.06	0.07	0.03	0.06
2620 *T_ratio_*	3.14	1.73	3.97	1.22	2.50
SD	0.06	0.03	0.08	0.02	0.05
2600 *T_ratio_*	2.79	1.78	2.30	1.27	1.62
SD	0.06	0.04	0.05	0.03	0.03

* no significant difference (at significant level α = 0.05), SD—standard deviation.
